# Advances in Neuropathic Pain Research: Selected Intracellular Factors as Potential Targets for Multidirectional Analgesics

**DOI:** 10.3390/ph16111624

**Published:** 2023-11-17

**Authors:** Katarzyna Ciapała, Joanna Mika

**Affiliations:** Department of Pain Pharmacology, Maj Institute of Pharmacology, Polish Academy of Sciences, 12 Smetna Str., 31-343 Kraków, Poland; kat.ciapala@gmail.com

**Keywords:** neuropathic pain, minocycline, astaxanthin, fisetin, peimine, p38MAPK, ERK, JNK, NF-κB, PI3K, NRF2

## Abstract

Neuropathic pain is a complex and debilitating condition that affects millions of people worldwide. Unlike acute pain, which is short-term and starts suddenly in response to an injury, neuropathic pain arises from somatosensory nervous system damage or disease, is usually chronic, and makes every day functioning difficult, substantially reducing quality of life. The main reason for the lack of effective pharmacotherapies for neuropathic pain is its diverse etiology and the complex, still poorly understood, pathophysiological mechanism of its progression. Numerous experimental studies, including ours, conducted over the last several decades have shown that the development of neuropathic pain is based on disturbances in cell activity, imbalances in the production of pronociceptive factors, and changes in signaling pathways such as p38MAPK, ERK, JNK, NF-κB, PI3K, and NRF2, which could become important targets for pharmacotherapy in the future. Despite the availability of many different analgesics, relieving neuropathic pain is still extremely difficult and requires a multidirectional, individual approach. We would like to point out that an increasing amount of data indicates that nonselective compounds directed at more than one molecular target exert promising analgesic effects. In our review, we characterize four substances (minocycline, astaxanthin, fisetin, and peimine) with analgesic properties that result from a wide spectrum of actions, including the modulation of MAPKs and other factors. We would like to draw attention to these selected substances since, in preclinical studies, they show suitable analgesic properties in models of neuropathy of various etiologies, and, importantly, some are already used as dietary supplements; for example, astaxanthin and fisetin protect against oxidative stress and have anti-inflammatory properties. It is worth emphasizing that the results of behavioral tests also indicate their usefulness when combined with opioids, the effectiveness of which decreases when neuropathy develops. Moreover, these substances appear to have additional, beneficial properties for the treatment of diseases that frequently co-occur with neuropathic pain. Therefore, these substances provide hope for the development of modern pharmacological tools to not only treat symptoms but also restore the proper functioning of the human body.

## 1. Neuropathic Pain

According to the definition formulated by the International Association for the Study of Pain (IASP), neuropathic pain is caused by somatosensory nervous system damage or disease and affects approximately 7–10% of the population [1,2]. It is manifested by abnormal sensations (dysesthesia) or pain caused by physiologically painless stimuli (allodynia) of a continuous or paroxysmal nature. People affected by this type of pain exhibit symptoms such as burning, tingling or stabbing pain [1]. These symptoms significantly reduce quality of life and have a negative impact on the physical, emotional, and social aspects of human functioning. Central neuropathic pain occurs in spinal cord injuries and multiple sclerosis, and sometimes accompanies strokes. Apart from diabetes (diabetic neuropathy) and other conditions associated with metabolic disorders, the most common causes of painful peripheral neuropathies are shingles; neuropathies associated with HIV infection; nutritional deficiencies; toxins; and genetic and immunological diseases [3]. This type of pain is also common in cancer, not only as a direct effect of the tumor on the peripheral nerves, but also a side effect of some chemotherapeutic agents or as a result of damage caused by radiation [4]. Neuropathic pain is distinguished by a reduced sensitivity to treatment with conventional painkillers; weaker effects of the two main groups of analgesics, opioids and nonsteroidal anti-inflammatory drugs, are observed. Therefore, its therapy requires a more comprehensive and interdisciplinary approach [1]. Currently, pregabalin and gabapentin, including extended-release formulations, as well as tricyclic antidepressants, are recommended as first-line drugs; second-line drugs are patches with capsaicin, lidocaine or tramadol; and third-line drugs are opioids and botulinum toxin type A [3].

Despite intensive research into neuropathic pain mechanisms, most available pharmacological treatments are based on old medicaments or drugs designated for other therapeutic interventions. Importantly, according to statistics, less than half of patients suffering from neuropathic pain report actual pain relief [2], which indicates the need for new drugs and more effective therapies for this condition. The latest data in the literature, including ours, suggest that, in addition to substances directed against one specific target, compounds with a broader spectrum of action may have analgesic properties. This is understandable when taking into consideration the complexity of neuropathic pain patomechanisms. For years, the mitogen-activated protein kinase (MAPK) family has been considered as one of the most important intracellular signaling pathways influencing nociceptive transmission in neuropathic pain. Initially, researchers focused on neurons, but in the last two decades, many studies have pointed out the important role of MAPKs in glial cells [5,6,7,8,9,10,11,12,13,14,15,16]. The MAPK family consists of three members, p38, extracellular signal-regulated kinase (ERK), and c-Jun N-terminal kinase (JNK); they are components of a series of crucial signal transduction pathways that regulate processes such as embryogenesis, cell differentiation, cell proliferation, and cell death [17]. MAPKs have a hierarchical structure, and consist of MAPK class kinases that are phosphorylated and activated by MAPKK (or MAP2K), which are in turn phosphorylated and regulated by MAPKKK (or MAP3K). Typically, MAPKKKs are activated by interactions with proteins belonging to the family of small GTPase proteins such as Ras/Rap or RhoA [18]. In our opinion, MAPK kinases may be promising therapeutic targets in neuropathic pain, especially when comodulated with other factors involved in nociception. In the following paper, using the PubMed database (applied keywords: neuropathic pain, minocycline, astaxanthin, fisetin, peimine, p38MAPK, ERK, JNK, NF-κB, PI3K, and/or NRF2), we discuss some of the interesting multitarget substances that exert analgesic effects to draw attention to their possible usefulness for neuropathic pain management. Importantly, among these are dietary supplements that are relatively safe to use and naturally derived, which makes them exceptionally interesting for further investigations.

## 2. MAPKs and Neuropathic Pain

**p38 mitogen-activated protein kinases (p38 MAPKs)** are a family of serine/threonine kinases expressed by many cells and consist of four isoforms: p38α, p38β, p38γ and p38δ. It has already been shown in rodent studies that, after nerve injury, the activation of p38 takes place in spinal microglia but not in neurons or astrocytes [8,15,19,20]. It is well established that p38 contributes to the development of neuropathic pain and additionally influences opioid treatment effectiveness [15,19,20,21,22,23,24]. Many pharmacological studies have provided evidence that the intrathecal administration of selective p38 inhibitors (skepinone-L [25], SB203580 [15,19,26,27], FR167653 [24], and CNI-1493 [28]) diminish the development of neuropathic pain. Moreover, FR167653 improves morphine analgesia in diabetic mice [29], and that SB203580 delays morphine tolerance development in rats [30]. It was also shown that an intraperitoneal injection of the p38α MAPK inhibitor SD-282 reversed or prevented alterations in mechanical and thermal responses induced by prolonged hyperglycemia in diabetic rats [31]. Importantly, the first clinical trials were undertaken to evaluate the analgesic action of the potent p38 inhibitors, dilmapimod (SB-681323) [32] and losmapimod (GW856553) [33], in patients suffering from neuropathic pain. Dilmapimod appeared to be promising; however, it needs to be further evaluated in larger trials to precisely determine the potency of its analgesic effect. The involvement of p38 in nociception is still under investigation. Behavioral experiments showed that the recently discovered FGA-19, which acts as a docking-site-oriented p38 inhibitor, relieves inflammatory pain [34]. Additionally, a new pyridin-2(1H)one derivative was developed, which is a selective p38 inhibitor that alleviates mechanical hypersensitivity in inflammatory pain [35]. Both compounds are extremely interesting but have not yet been studied in the context of neuropathic pain. The experimental data provide evidence that p38 activation is initiated by the stimulation of NMDA receptors and participates in hyperalgesia [36]. In many neuropathic pain models, increased activation of p38MAPK is observed in microglia [11,19,24,37,38]. p38 MAPK is involved in the synthesis of many pronociceptive factors via transcriptional regulation [8,12,26,39,40,41]. It was shown that an increase in p38 levels in microglia enhances the synthesis of pronociceptive factors such as TNFα, IL-1β, IL-18, IL-6 and iNOS [8,42,43,44,45,46,47,48,49]. Interestingly, p38 activation in microglia was thought to play an equivalent role in both sexes; however, recently, it was shown that first, nerve injury primarily activates spinal p38 in male mice, and second, that an intrathecal injection of the selective p38 inhibitor skepinone reduced neuropathic pain in male but not in female rodents [25]. This is important information because clinical data clearly indicate that neuropathic pain develops much more often in women and is more difficult to treat [50]. The latest trends in research on the neuroimmune basis of neuropathic pain focus on sexual dimorphism, and the main difference between sexes is the activation of microglia.

**Extracellular signal-regulated kinases 1 and 2 (ERK1/2)** are serine/threonine kinases that also belong to the MAPK family. ERK1/2 kinases are involved in many cellular regulation processes and are important for nociceptive transmission [5,51,52,53,54]. ERK signaling can be altered by blocking the upstream protein kinases MEK1/2 (mitogen-activated protein kinase 1/2). ERK1/2 kinases affect the function of the A-type potassium channel Kv4.2 [55] and voltage-dependent calcium channels in sensory neurons [56]. However, ERK1/2 kinases are activated in both neurons and glia after nerve and spinal cord injury, which causes the development of hypersensitivity [8,16,21,57]. The available literature clearly indicates that ERK activation is crucial for the development of neuropathic pain symptoms because it causes the secretion of many pronociceptive factors, such as TNFα, IL-1β, iNOS, and nNOS [8,21,22,58]. It has already been shown that the intrathecal injection of selective MEK-ERK pathway inhibitors, such as U0126 [57,59], PD198306 [60], and PD98059 [8,16,22], reduces neuropathic pain symptoms in rodent models. Moreover, it has been demonstrated that PD98059 reduces nerve injury and elevates the levels of p38, ERK1/2, JNK, and pronociceptive factors such as IL-1beta, IL-6, and iNOS [22]. The latest research also indicates that repeated intrathecal injections of U0126 [59] and PD98059 [22] potentiate morphine and/or buprenorphine analgesia in rats with neuropathic pain. Moreover, PD98059 delays morphine tolerance development in rats [30]. Interestingly, it was recently shown, using an oxaliplatin-induced neuropathic pain model, that the activation of ERK1/2 kinases promotes the expression and activation of CREB, which leads to an increase in Nav1.6 protein expression, enhancing neuronal excitability and evoking pain [61]. The results of numerous studies clearly indicate the important role of the MEK/ERK signaling pathway in neuropathic pain.

**c-Jun N-terminal kinase (JNK)** is the third major member of the MAPK family, with three isoforms, JNK1, JNK2 and JNK3 [62]. JNKs have been revealed to be involved in neurodegeneration and inflammatory responses [7,62,63,64]. In mammals, the JNK1 and JNK2 proteins are ubiquitously expressed, whereas JNK3 is found almost exclusively in the brain [65]. The results show that JNK3 is present in neurons, but JNK1 is mainly expressed in nonneuronal cells, including immune cells [7,66,67]. JNK1 and JNK2 are expressed in the spinal cord [7]; however, JNK1 is predominantly upregulated in astroglial cells after nerve injury [7,22,68]. Compared to p38 and ERK, much less is known about how JNK regulates nociception. To date, JNK activation appears in DRGs in primary sensory neurons after nerve injury but only in the early phase of neuropathy (until day 10) [7]. In contrast, at the spinal cord level, JNK upregulation is observed in astrocytes over a long period of time. Therefore, it is suggested that this kinase is especially important with respect to the persistence of neuropathic pain. It was already shown that SP600125, a small-molecule inhibitor of JNK1/2/3, diminishes spinal nerve ligation-induced neuropathic pain [7] and prevents hypersensitivity induced by the antiretrovirals zalcitabine and stavudine in mice [69]. Similarly, the intrathecal administration of D-JNKI-1, JNK1/2/3 inhibitor, diminishes spinal nerve ligation-induced hypersensitivity by suppressing the phosphorylation of c-Jun in spinal astrocytes [7]. Moreover, the infusion of D-JNKI-1 into the L5 DRG prevented, but did not reverse, previously established tactile hypersensitivity [7]. In summary, JNK activation in the DRG and spinal cord plays distinct roles in regulating the development and maintenance of neuropathic pain, respectively. Targeting the JNK pathways in glia and sensory neurons may represent a novel effective treatment for intractable neuropathic pain symptoms.

In summary, taking into account both the literature and our many years of research, we think that, in the treatment of neuropathic pain, pharmacological tools with broad-spectrum actions are particularly valuable. In our review, we paid attention to substances that not only have a beneficial effect on kinases from the MAP family but also influence several nociceptive factors listed as targets in Table 1. We focused on four substances, briefly presenting their mechanism of action, impacts on nociceptive factors and effects on opioid treatment efficacy in neuropathy. In the following sections, we discuss in detail the importance of minocycline, astaxanthin, fisetin, and peimine in nociceptive transmission and present data on their analgesic effects on neuropathic pain. Additionally, we also include information about other properties of these substances that are not directly related to nociception but are useful from a clinical point of view.

## 3. MMPs and Neuropathic Pain

Recently, there has been an increasing amount of new information about the important role of matrix metalloproteinases (MMPs) in the development of neuropathy. MMPs are involved in many proteolytic processes that require matrix remodeling in both physiological and pathological conditions. A previous study showed that MMPs were strongly activated after CNS and PNS injury, contributing to disruption of the blood–brain barrier and enabling the influx of immune cells into the nervous system [101]. Numerous studies indicate that MMP-9 and MMP-2 are involved in nociception. In 2008, it was documented for the first time that L5 spinal nerve ligation induces a quick (24 h after injury) rise in MMP-9 levels in the DRGs of rats. The authors also showed that the level of MMP-2 did not increase until day 7, but lasted up to 21 days [102]. The published results suggest that MMP-9 is responsible for the initiation, while MMP-2 is responsible for the maintenance, of neuropathic pain [102]. Later, it was described that sciatic nerve injury also strongly enhanced the levels of MMP-9 and TIMP-1 (tissue inhibitor of metalloproteinase 1) not only in DRGs but also within the spinal cord [26]. Moreover, it was reported that after axon damage, Schwann cells release a large amount of MMP-9, which contributes to the initiation of macrophage infiltration and degradation of myelin basic protein [103,104]. As a consequence, neuron hypersensitivity occurs, and sensitization develops [105].

Importantly, pharmacological studies conducted in parallel confirmed the important role of MMP-9 in the development of neuropathic pain symptoms. It has been suggested that an intrathecal injection of MMP-9 evokes neuronal hypersensitivity by transforming inactive proIL-1beta to the active IL-1beta form [102]. As a result, there is increased excitability of neurons and uncontrolled activation of microglia in the dorsal horn of the spinal cord [26,102].

Notably, MMP-2 also cleaves IL-1β; however, it activates astrocytes at later time points [102]. Moreover, it was revealed that both the gene deletion of MMP-9 or MMP-9 siRNA administration reduced the development of hypersensitivity in animal models of neuropathic pain [101,102] Similarly, the preemptive and repeated administration of MMP-9 inhibitor-I for five days after nerve injury delayed hypersensitivity development [102]. Furthermore, the intrathecal administration of antibodies against IL-1beta reduces MMP-9-induced hypersensitivity [102]. Additionally, the administration of TIMP-1 diminishes the development of neuropathy to a similar extent [102]. The results obtained by many researchers indicate that time-dependent changes in MMP-2 and MMP-9 levels offer different options for the treatment of neuropathic pain [102], and that blocking MMP-9 appears to be effective in inhibiting the development of hypersensitivity.

## 4. MINOCYCLINE—A MAPK and MMP Modulator in Neuropathic Pain

Considering the important role of MAPKs and MMPs in the development of neuropathic pain, it can be assumed that substances which affect these factors will also influence nociception. Previous studies have identified minocycline, a second-generation tetracycline antibiotic that acts against both Gram-positive and Gram-negative bacteria, as one of these substances [106,107,108,109]. Minocycline is a highly lipophilic molecule that can easily pass through the blood–brain barrier [108,110], which enables its use in the treatment of many diseases [107,108,111,112,113,114,115,116,117,118,119,120,121,122,123,124,125]. Numerous data in the literature indicate that, although minocycline exhibits effects characteristic of antibiotics, it also exerts other biological properties, such as anti-inflammatory, antioxidant, antiapoptotic and immunomodulatory properties [106]. What is more, minocycline also plays a neuroprotective role, which has been confirmed in experimental models of ischemia [123,124], brain [125] and spinal cord [111,112,122] injury, and several neurodegenerative conditions [113,114,115,116,117]. Moreover, preclinical data have confirmed the ability of minocycline to prevent tumor growth [126] and human immunodeficiency virus replication [127].

Importantly, numerous studies have indicated that minocycline reduces neuropathic pain symptoms not only in animal models [26,74,75,81,128,129,130] but also in humans [131]. In 2006, Piao et al. showed that the molecular mechanism of minocycline’s action is the inhibition of p38MAPK in microglia, while in 2013, Niimi et al. provided evidence of its ability to inhibit the activity of MMP-9 [73]. The crucial role of both these molecular targets of minocycline in neuropathic pain is beyond doubt. It was already shown that repeated intraperitoneal and intrathecal minocycline administration strongly diminished microglial activation [11,26,37] by reducing the activation of p38 kinase [11,42]. The inhibition of microglial activation has very important consequences, since these cells have already been shown to be the source of many factors with pronociceptive properties [132,133]. It has already been demonstrated that repeated minocycline administration downregulates pronociceptive factors (IL-6 and IL-18) but does not influence antinociceptive factors (IL-1α, IL-4, and IL-10) [26]. Moreover, in vitro studies showed that minocycline reduces the activation of M1-polarized microglia but does not affect the beneficial M2-polarized microglia [26,103]. The critical role of MMP-9 in the development of neuropathic pain was also confirmed in animal models, as described above. Interestingly, the MMP-9 inhibitor (MMP9-INH. I) was more effective at diminishing neuropathic pain symptoms than a selective p38MAPK inhibitor (SB203580) [26]. Therefore, in our opinion, the excellent analgesic effect of minocycline is related to the direct inhibition of both MMP-9 and p38MAPK, and consequently, after repeated administration, it indirectly affects the levels of spinal pronociceptive factors (IL-1β [82] IL-6 [26]; IL-18 [26]; iNOS [81]; XCL1 [83], MMP-2 [26]) and kinases (pERK [72,84]; PI3K [84]). Importantly, previous studies have revealed that preemptive and repeated intrathecal or intraperitoneal minocycline administration potentiated morphine analgesia [75,130]. Similar results were obtained after intrathecal coadministration of minocycline with selective ligands of the MOP (DAMGO), KOP (U50,488H) and NOP (nociceptin) receptors [129,130]. Notably, minocycline coadministered with morphine strongly delayed the development of morphine tolerance [74]. Its beneficial effect is probably related to the ability of this substance to silence microglial activation, which reduces the production of pronociceptive factors that are capable of exhibiting anti-opioid activity, as suggested by many studies [22,132,133,134,135,136,137,138,139,140,141,142]. These newly discovered properties of minocycline are very promising from a clinical point of view, as there is still a need for drugs that improve the effectiveness of opioid treatment in patients suffering from neuropathic pain, and that inhibit or delay the development of tolerance to its analgesic effect. The latest data suggest that minocycline has beneficial effects on relieving diabetes-induced or chemotherapy-induced neuropathic pain in humans [131]. Of particular interest are studies indicating that, apart from hypersensitivity reduction, minocycline in a rat model of streptozotocin-induced diabetic neuropathy also has a beneficial effect on cardiodynamic parameters [143] and inhibits kidney damage [144].

**Scheme 1:**
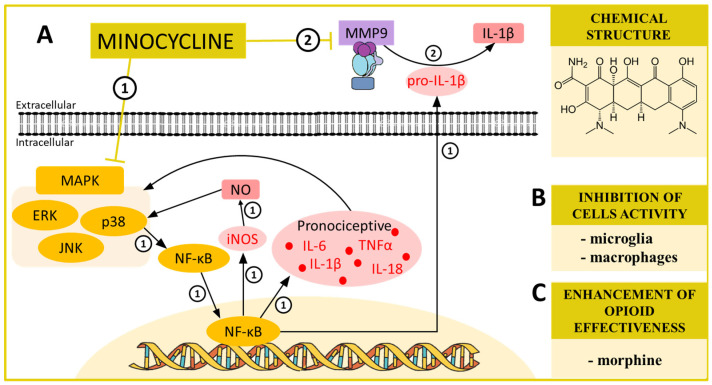
(**A**) **The possible mechanisms of minocycline action as indicated by various *in vivo*** ➀➁ **and *in vitro*** ➀➁ **studies:** ➀ inhibition of MAP kinases by minocycline in turn indirectly inhibits NF-κB, and as a consequence, the production of pronociceptive cytokines such as IL-6, IL-1β, IL-18 and TNFα [26,82] and also evokes the inhibition of the inducible nitric oxide synthase (iNOS) production, which prevents p38 activation by nitric oxide (NO) [81]—blockade of the synthesis of pronociceptive factors may be the cause of the analgesic effect of minocycline; ➁ inhibition of MMP9, which attenuates the transformation of inactive proIL-1β to the active, pronociceptive IL-1β [73,102]. (**B**) **The influence of minocycline on activation of cells important for neuropathic pain development as proposed by *in vitro*** [26,83] **and *in vivo*** [26,83] **studies; and** (**C**) **the influence of minocycline on opioid effectiveness in neuropathic pain models** [74]. ***Abbreviations***: mitogen-activated protein kinases (MAPK); extracellular signal-regulated kinase (ERK); p38 mitogen-activated protein kinase (p38); c-Jun N-terminal kinase (JNK); nuclear factor kappa B (NF-κB); inducible nitric oxide synthase (iNOS); nitric oxide (NO); matrix metalloproteinase 9 (MMP9); tumor necrosis factor α (TNFα); interleukin 1 beta (IL-1β); interleukin 6 (IL-6); interleukin 18 (IL-18).

In summary, minocycline restores the neuroimmunological balance that is biased toward pronociceptive factors in the development of neuropathic pain. The proposed mechanisms by which minocycline produces its analgesic effects are presented in brief in Scheme 1. Numerous studies provide a rational basis for the further evaluation of minocycline in the treatment of neuropathic pain. A safe translation from animal studies to clinical trials is still challenging. Although minocycline is a well-tolerated drug it exhibits some side effects [120,145,146]; therefore, more studies are still needed.

## 5. NRF2 and Neuropathic Pain

Recent studies have highlighted the potential role of the nuclear factor erythroid 2-related factor 2 (NRF2) pathway in the modulation of neuropathic pain. One of the mechanisms implicated in the pathogenesis of neuropathic pain is oxidative stress, characterized by an imbalance between reactive oxygen species (ROS) production and antioxidant defense mechanisms. NRF2 is a transcription factor that plays a crucial role in cellular defense against oxidative stress [147]. Under normal conditions, NRF2 is localized in the cytoplasm and bound to Kelch-like ECH-associated protein 1 (KEAP1) [147]. NRF2 is the product of the NFE2L2 gene and a member of the cap ‘n’ collar subfamily of basic-leucine zipper (bZip) transcription factors [148]. NRF2 contains a bZip domain at the C-terminus that is responsible for the formation of heterodimers with other bZip proteins [149]. In response to oxidative stress, NRF2 dissociates from KEAP1, translocases into the nucleus, and by these heterodimers influences approximately 250 human genes located at the regulatory enhancer sequence known as the antioxidant response element (ARE), leading to the upregulation of various antioxidant and cytoprotective enzymes. Studies have shown that NRF2 activation exerts a neuroprotective effect [150].

In animal models of neuropathic pain, NRF2 activation has been shown to attenuate hypersensitivity and reduce neuronal damage [151,152]. This effect is mediated through the upregulation of antioxidant enzymes, such as heme oxygenase-1 (HO-1), superoxide dismutase (SOD), and glutathione peroxidase (GPx), which scavenge ROS and protect neurons from oxidative damage. Additionally, NRF2 activation inhibits the production of proinflammatory cytokines, such as TNF-α and IL-1β, which contribute to neuropathic pain hypersensitivity [153]. Interestingly, pharmacological, and genetic research reports crosstalk between NF-κB and NRF2, such that the absence of NRF2 can exacerbate NF-κB activity, leading to increased cytokine production. Moreover, NF-κB can modulate the transcription and activity of NRF2 [154]. In our opinion, understanding these relationships in experimental models should provide insights into the development of pharmacological tools and more effective therapies for neuropathic pain.

Only a few selective NRF2 modulators have been studied in animal models of neuropathy to date. One of these substances is sulforaphane, a natural compound found in cruciferous vegetables such as broccoli and cabbage [155]. It has been shown to activate NRF2 and exert neuroprotective effects [156]. Studies have demonstrated that sulforaphane administration can reduce neuropathic pain behaviors in animal models by reducing oxidative stress [157,158]. The latest published data indicate that sulforaphane also increases the effectiveness of morphine treatment in neuropathy [157]. Another NRF2 modulator is dimethyl fumarate, which was recently shown to reverse hypersensitivity induced by nerve damage in male and female mice by reducing the injury-induced elevation in IL-1β, CCL2 and TNFα levels [159]. The advantage of dimethyl fumarate is that it is already approved by the FDA for the treatment of multiple sclerosis. Therefore, clinical trials could be initiated to determine its usefulness in the treatment of neuropathic pain of various etiologies [160]. Dimethyl fumarate activates NRF2 by covalently modifying KEAP1, leading to NRF2 release and the subsequent upregulation of antioxidant enzymes [161]. Preclinical studies have shown that dimethyl fumarate administration can alleviate neuropathic pain symptoms by reducing oxidative stress and modulating neuroinflammatory responses [159,162]. Bardoxolone methyl, which is also an activator of NRF2, showed promising results in preclinical studies in the treatment of neuropathic pain caused by nerve damage [90] and diabetes [90,163]. Moreover, the administration of morphine, buprenorphine and oxycodone preceded by the injection of bardoxolone-methyl effectively alleviated tactile and thermal hypersensitivity after sciatic nerve injury [90]. Taking into account the available literature and our own studies, we believe that, from a clinical point of view, the regulation of NRF2 in neuropathy is extremely important, and that pharmacological modulation of this factor may contribute to more effective polytherapy.

## 6. ASTAXANTHIN—A MAPK and NRF2 Modulator in Neuropathic Pain

Taking into consideration the substantial influence of MAPKs and NRF2 on neuropathic pain development, it can be expected that their modulators would exert excellent analgesic properties. The available literature demonstrates that astaxanthin, a naturally occurring carotenoid that belongs to the xanthophyll family, which is found in various marine organisms, such as microalgae, krill, shrimp, and salmon, is one of these modulators. Astaxanthin is known for its potent antioxidant activity and has been shown to be significantly more effective than other well-known antioxidants, such as vitamin C, vitamin E, and beta-carotene [164]. The molecular structure of astaxanthin enables it to neutralize free radicals and protect cells from oxidative damage and to reduce the expression levels of pronociceptive factor genes [165]. This makes it potentially beneficial for conditions associated with chronic inflammation, such as arthritis, cardiovascular diseases, and neurodegenerative disorders [166].

**Scheme 2:**
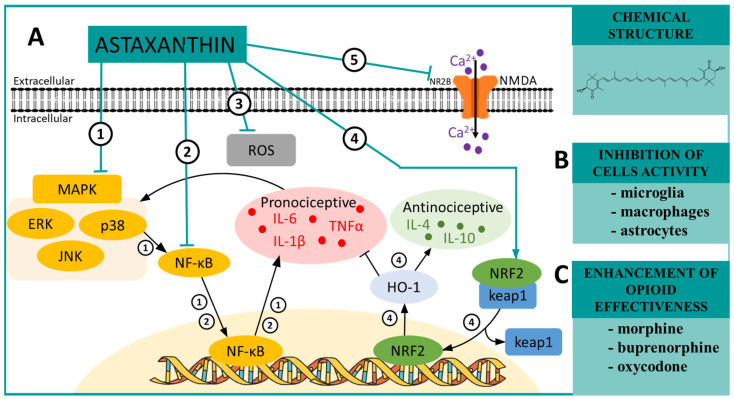
(**A**) **The possible mechanisms of astaxanthin action as indicated by various *in vivo*** ➀➁➃➄ **and *in vitro*** ➂ **studies:** ➀ inhibition of MAP kinases by astaxanthin, which in turn indirectly inhibits NF-κB, and as a consequence, the production of pronociceptive cytokines, such as IL-6, IL-1β [85], is lowered; ➁ inhibitory effect on NF-κB, which causes a decrease in the synthesis of the aforementioned pronociceptive factors [86]; ➂ inhibition of the ROS production, which are involved in maintenance of persistent pain [88]; ➃ activation of NRF2, which causes the dissociation of keap1 with translocation of NRF2 to the nucleus, and as a consequence, an increase in HO-1 synthesis, which evokes reduction of pronociceptive factors levels, and simultaneously increases the production of antinociceptive cytokines [171]; ➄ inhibition of the NR2B, NMDA receptor subunit, which, as a consequence, reduces Ca^2+^ mobilization and diminishes hypersensitivity [87]. (**B**) **The influence of astaxanthin on the activation of cells important for neuropathic pain development as proposed by *in vitro*** [85] **and *in vivo*** [85] **studies; and** (**C**) **the influence of astaxanthin on opioid effectiveness in a neuropathic pain model** [90]. ***Abbreviations:*** mitogen-activated protein kinases (MAPK); extracellular signal-regulated kinase (ERK); p38 mitogen-activated protein kinase (p38); c-Jun N-terminal kinase (JNK); nuclear factor kappa B (NF-κB); tumor necrosis factor α (TNFα); interleukin 1 beta (IL-1β); interleukin 6 (IL-6); interleukin 4 (IL-4); interleukin 10 (IL-10); nuclear factor erythroid 2-related factor 2 (NRF2); Kelch-like ECH-associated protein 1 (keap1); heme oxygenase (HO-1); N-methyl-D-aspartate receptor (NMDA); NMDA receptor subunit epsilon-2 (NR2B); reactive oxygen species (ROS).

A study from 2018 evaluated the analgesic effect of trans-astaxanthin following three weeks of oral administration in mice exposed to sciatic nerve injury [167]. This substance not only ameliorated symptoms of thermal and mechanical hypersensitivity but also reduced depressive-like behaviors induced by long-term neuropathic pain. Additionally, it revealed that the analgesic action of astaxanthin is relatively fast, which may be due to its ability to cross the blood–brain and blood–spinal cord barriers, and even more interestingly, these pain-relieving effects were observed as quickly as four days after the last administration [167]. This effect is a result of its beneficial influence on the serotonergic system and the kynurenine pathway, and the downregulation of pronociceptive cytokines such as IL-1β, IL-6 and TNFα in the spinal cord in mice after sciatic nerve injury [167]. A subsequent study performed in rats following compression spinal cord injury revealed that an intrathecal injection of astaxanthin attenuated neuropathic pain development and reversed motor dysfunction, which is associated with a reduction in elevated levels of p-p38 MAPK, NR2B and TNFα in the spinal cord [87]. The analgesic effect of astaxanthin was also confirmed in other studies conducted on rats after spinal cord injury, where protein analysis showed decreased activation of ERK1/2 and increased activation of AKT after treatment with this drug [168]. Moreover, an additional mechanism by which astaxanthin may exert an analgesic effect is its influence on NMDA receptors. In silico molecular docking studies revealed that astaxanthin fits into the inhibitory binding pocket of the NMDA receptor, and especially into that of NR2B, a subunit important for nociception [89]. It was also shown that, using in vitro studies on C6 glial cells, astaxanthin administration reduced LPS-evoked inflammation by decreasing the activation of astrocytes, which are known to be important in the progression of neuropathic pain [89]. Recent research performed on spinal nerve ligation-exposed mice also confirms the analgesic properties of astaxanthin. The study found that astaxanthin could effectively inhibit neuroinflammation by reducing p-ERK1/2 and p-p38 MAPK activation and the nuclear translocation of NF-κB p65 [85]. In recent years, numerous studies have suggested that a very potent target of astaxanthin is NRF2. It was shown that astaxanthin upregulates the expression of NRF2-dependent antioxidant enzymes and phase II detoxifying enzymes, which help combat oxidative stress and inflammation [169,170]. In complete Freund’s adjuvant-induced inflammatory pain in mice, astaxanthin relieved mechanical and thermal hypersensitivity and inhibited the inflammatory response [171]. Although the anti-inflammatory, antioxidative and anti-neuropathic effects of astaxanthin have been previously highlighted, its peripheral antinociceptive mechanisms are not fully understood. Newer studies also suggest the involvement of the l-arginine/NO/cGMP/K_ATP_ pathway in the analgesic effect of astaxanthin [172]. Moreover, it was also reported that the intrathecal administration of astaxanthin in mice subjected to sciatic nerve injury increased the effectiveness of morphine, buprenorphine and oxycodone [90]. Importantly, in 2023, the results of a multicenter clinical trial were published, showing that the drug FlexPro MD^®^, containing astaxanthin, is well tolerated and can be effectively used to treat pain in patients with osteoarthritis [173]. Additionally, there are data that suggest that astaxanthin may influence different states that sometimes co-occur with neuropathic pain and are related to its etiology; for example, it has preventive effects against diabetes, as indicated by lower glucose levels after supplementation with this substance [174,175]. There are also initial reports indicating that astaxanthin can block the proliferation of cancer cells, which is also a promising prognostic, because neuropathic pain often develops as a result of tumors [176,177]. What is more, this substance also exerts neuroprotective [178] and cardioprotective [179] properties. The suggested targets affected by astaxanthin are presented in brief in Scheme 2. Further research, primarily preclinical research, should be conducted to determine the usefulness and mechanism of action of astaxanthin in neuropathies of various etiologies.

## 7. NF-κB and Neuropathic Pain

Nuclear factor kappa B (NF-κB) is a pleiotropic protein transcription factor that is activated in response to a wide variety of immune stimuli [180,181]. Recently, numerous papers have indicated an essential role of the NF-κB pathway in pain [21,22,44,59,182,183,184]. In the central nervous system, NF-κB primarily exists as a p50/p65 heterodimer [180,185,186] and its activation is mediated via two distinct kinase-dependent pathways, classical (canonical) and alternative (noncanonical). For nociception, the classical pathway seems to be the most important since it is initiated by many receptors, including cytokine receptors and TLRs. [187,188].

It is currently well established that after nerve damage, activated NF-κB influences the expression of many genes, which contributes to the production of many nociceptive factors [21,119,182,184,189]. Moreover, increased NF-κB activity has been demonstrated not only in the DRGs but also in the spinal cord of various neuropathic pain model animals [21,22,44,182,184,190,191]. Recently, it was also shown that the levels of the pronociceptive factors TNFα and IL-6, which are involved in neuropathic pain development after spinal cord injury, can be decreased by inhibiting the TLR4/MyD88/NF-κB pathway [192]. Similarly, after sciatic nerve injury, the selective inhibition of NF-κB reduces hypersensitivity, which has been shown to be associated with beneficial effects on the levels of the pronociceptive factors IL-6 and iNOS [182]. Interestingly, repeated intrathecal administration of parthenolide (an NF-κB inhibitor) increases microglial activation after sciatic nerve injury in rats [21], although it provides pain relief and increases the analgesic properties of morphine [21,59]. These surprising results were partly explained by in vitro studies, in which it was shown that parthenolide, apart from activating microglia, reduces the levels of pronociceptive factors produced by M1 microglia (iNOS, IL-1β, IL-18) and increases the levels of antinociceptive factors produced by M2 microglia (TIMP1, IL-10) [21,59]. It is already known that parthenolide directly downregulates NF-κB and probably indirectly modulates p38 and ERK1/2 kinase [21,59]. In conclusion, the available literature indicates that the inhibition of NF-κB may directly or indirectly alleviate neuropathic pain symptoms and promote neuroprotective microglial M2 polarization.

## 8. PEIMINE—A MAPK and NF-κB Modulator in Neuropathic Pain

**Scheme 3:**
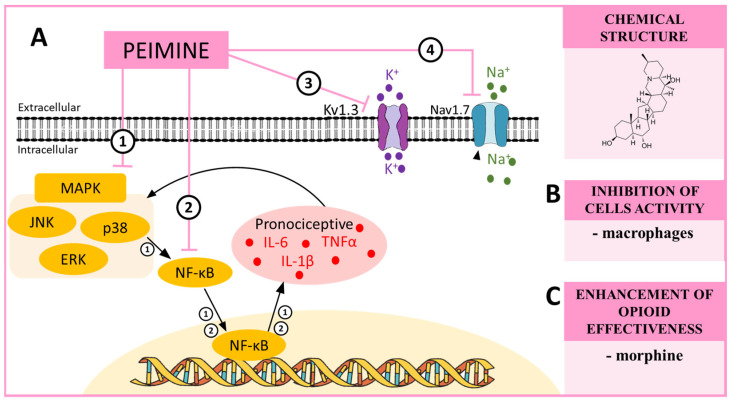
(**A**) **The possible mechanisms of peimine action as indicated by various *in vitro*** ➀➁➂➃ **studies:** ➀ inhibition of MAP kinases by peimine, which in turn inhibits NF-κB, and, as a consequence, the production of pronociceptive cytokines such as IL-6 or IL-1β [91]; ➁ inhibitory effects of NF-κB, which causes a decrease in the synthesis of pronociceptive factors [195]; ➂ inhibition of the Kv1.3 potassium channels, which are involved in the maintenance of persistent pain [93]; ➃ inhibition of the Nav1.7 sodium channels, which are involved in the development of hypersensitivity [93]. (**B**) **The influence of peimine on the activation of cells important for neuropathic pain development as proposed by *in vitro* studies** [199]; and (**C**) **the influence of peimine on opioid effectiveness in neuropathic pain models** [90]. ***Abbreviations:*** mitogen-activated protein kinases (MAPK); extracellular signal-regulated kinase (ERK); p38 mitogen-activated protein kinase (p38); c-Jun N-terminal kinase (JNK); nuclear factor kappa B (NF-κB); tumor necrosis factor α (TNFα); interleukin 1 beta (IL-1β); interleukin 6 (IL-6).

A very interesting substance in terms of its potential analgesic effect and its ability to targets MAPKs and NF-κB is peimine. It is a natural compound that belongs to a class of substances known as amaryllidaceae alkaloids. It is primarily found in plants of the Amaryllidaceae family, such as the Chinese herb *Fritillaria thunbergii* [193]. This substance has been studied for its various biological activities, including its anti-inflammatory, antioxidant, antitumor, and antiviral properties [194]. It has been shown that peimine is able to inhibit both the growth of cancer cells and inflammation in animal models [195,196]. In Chinese medicine, it has been claimed to promote lung infection recovery, reduce phlegm, and relieve cough symptoms [194]. There are only a few studies on the pain-relieving effects of this compound. It was shown that peimine inhibits the writhing reaction induced by acetic acid in mice [197] and strongly reduces hypersensitivity to mechanical and thermal stimuli in mice after sciatic nerve injury [90]. Moreover, the administration of peimine with opioids resulted in more effective attenuation of tactile hypersensitivity in the case of morphine and oxycodone, while thermal hypersensitivity was more effectively reduced in the case of administration with morphine or buprenorphine [90]. The mechanism of the analgesic action of peimine in neuropathic pain has not yet been fully elucidated but it may be related to its beneficial effect on MAPKs and NF-κB. Moreover, emerging studies of peimines conducted in vitro indicate a very broad spectrum of activity. It has been shown that this substance can not only block the Nav1.7 ion channel but also preferentially inhibit the Kv1.3 ion channel [93], which are channels closely involved in nociceptive transmission. It was also recently pointed out that peimine targets nAChRs with high affinity, which might account for its anti-inflammatory actions [198]. The in vitro experiments performed on chondrocytes provide evidence that peimine can inhibit the IL-1β-induced activation of MAPK [91]. Moreover, a study on human mast cells proved that peimine reduced MAPK phosphorylation, downregulated nuclear NF-κB expression, and consequently inhibited the production of pronociceptive cytokines such as IL-6, IL-8, and TNFα [195]. Peimine significantly inhibits the phosphorylation of p38, ERK and JNK and decreases p65 and IκB in lipopolysaccharide-stimulated RAW 264.7 macrophages [92]. The suggested mechanisms of action of peimine are presented in brief in Scheme 3. Notably, the pharmacokinetics of peimine are sex-dependent; this drug is slowly eliminated from the plasma of male but not female Sprague–Dawley rats, and there are also substantial gender-related differences in pharmacokinetic parameters [194]. 

Undoubtedly, further research on peimine is needed to assess its usefulness in the treatment of neuropathic pain; however, according to the latest reports, this substance may have a toxic effect on the heart [194]. Importantly, we do not know whether the doses of peimine that could produce an analgesic effect will cause any side effects. At this point, this substance has enormous therapeutic potential that needs to be further explored.

## 9. PI3K/AKT and Neuropathic Pain

Phosphoinositide 3-kinase (PI3K) is a lipid kinase that modulates cell differentiation, proliferation, migration, and death by activating the downstream target protein kinase B, known as AKT [200,201]. Importantly, disorders in the functioning of the PI3K/AKT pathway have already been demonstrated in many diseases, including cancer, diabetes, cardiovascular diseases and neurological diseases [202]. Research conducted over the years shows that PI3K is closely involved in long-term potentiation of synaptic plasticity [203,204,205], while more recent studies clearly suggest its important role in nociceptive transmission [206,207,208,209,210]. Evidence indicates that PI3K and AKT are crucial mediators that lead to the activation of NF-κB, which plays an important role in the production of nociceptive factors (e.g., IL-1β, TNFα) in neuropathic pain [211]. Several pharmacological studies have indicated that the blockade of PI3K reduces hypersensitivity in animal pain models of inflammatory and neuropathic pain [206,207,209,212,213]. Intrathecal injection of PI3K and AKT inhibitors (wortmannin, LY294002, deguelin) started before L5 spinal nerve ligation reduced mechanical and thermal hypersensitivity in rats [207]. There are also reports showing that repeated treatment with LY294002 attenuates hypersensitivity in rats with painful diabetic neuropathy [214]. Surprisingly, after sciatic nerve injury in mice, it was shown that a single intrathecal administration of a selective PI3K activator, namely, 740 Y-P, decreased mechanical and thermal hypersensitivity on day 7 [90]. Moreover, single intrathecal administration of 740 Y-P combined with opioids (morphine, buprenorphine, oxycodone) relieves thermal, but not mechanical, hypersensitivity [90]. Further research is required to explain the molecular mechanism of the observed effects and the above-discussed discrepancies in the literature. In vitro studies have shown that the inhibition of the LPS-activated PI3K/AKT pathway in microglia diminishes the biosynthesis of pronociceptive factors [215,216]. Additionally, it was also shown that PI3K influences the morphine-induced migration of microglia [217]. Taking into account the available studies in the literature, it can be said with certainty that the role of the PI3K/AKT pathway in the central nervous system is ambiguous and appears to be dualistic. For example, after spinal cord injury, its activation prevents oxidative stress, inflammation and cell death, while its inhibition prevents the formation of glial scars [218]. The roles and mechanism of action of the PI3K/Akt pathway in the development of neuropathic pain evoked by nerve or spinal cord injury and diabetes, and its involvement in opioid treatment effectiveness, still need to be explained since the literature clearly suggests that this pathway has therapeutic potential.

## 10. FISETIN—A MAPK, NF-κB and PI3K Modulator in Neuropathic Pain

**Scheme 4:**
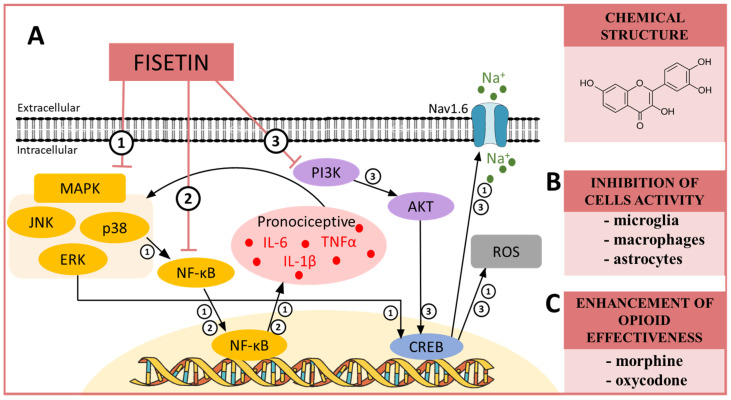
(**A**) **The possible mechanisms of fisetin action as indicated by various *in vivo*** ➀➁ **and *in vitro*** ➂ **studies:** ➀ inhibition of MAP kinases by fisetin, which in turn indirectly inhibits NF-κB, and, as a consequence, the production of pronociceptive cytokines, such as IL-6, IL-1β and TNFα [100], is lowered;➁ influence on NF-κB activation, which causes a decrease in the synthesis of pronociceptive factors [97]; ➂ inhibition of PI3K/AKT, which in turn influence the cAMP-response element binding protein (CREB) that impacts the expression of Nav1.6 sodium channels, which reduces hypersensitivity but also decreases the production of ROS, which are confirmed to be responsible for the maintenance of neuropathic pain [61,96,233]. (**B**) **The influence of fisetin on the activation of cells important for neuropathic pain development as proposed by *in vitro* studies** [94,234]; and (**C**) **the influence of fisetin on opioid effectiveness in neuropathic pain model** [90]. ***Abbreviations:*** mitogen-activated protein kinases (MAPK); extracellular signal-regulated kinase (ERK); p38 mitogen-activated protein kinase (p38); c-Jun N-terminal kinase (JNK); nuclear factor kappa B (NF-κB); tumor necrosis factor α (TNFα); interleukin 1 beta (IL-1β); interleukin 6 (IL-6); reactive oxygen species (ROS); phosphoinositide 3-kinase (PI3K); protein kinase B (AKT); cAMP response element-binding protein (CREB).

Fisetin, a substance with wide therapeutic potential, which includes effects on MAPK, NF-κB and PI3K, is a naturally occurring flavonoid compound found in various fruits and vegetables such as strawberries, apples, and onions [219]. Fisetin administration has been shown to have beneficial effects in preclinical animal models of diseases such as Alzheimer’s disease, Parkinson’s disease, Huntington’s disease, dementia, schizophrenia, stroke, traumatic brain injury, and the aging process [220,221,222,223,224,225,226]. This substance evokes a variety of pharmacological effects, such as anti-allergic, cancer-preventive and neuroprotective actions [227,228,229]. In 2015, it was shown that fisetin administration after oral drug treatment relieved thermal hypersensitivity in mice exposed to sciatic nerve injury. Importantly, this effect was a reversing action (not a prophylactic one) since the administrations started two weeks following injury, which is when pain-related behavior was observed. In this study, however, a reduction in mechanical hypersensitivity was not observed [98]. Interestingly, subsequent studies showed that after the intrathecal administration of fisetin in mice post sciatic nerve injury, both thermal and mechanical hypersensitivity were significantly reduced, and fisetin also improved the analgesic effect of morphine or oxycodone [90]. It was also confirmed by others that fisetin oral administration can delay diabetic neuropathic pain-related behavior in mice [99]. Although this chronic administration of fisetin did not affect the symptoms of hyperglycemia in diabetic mice, it reduced severe oxidative stress in the spinal cord, DRGs, and sciatic nerve. The analgesic properties of fisetin can be explained not only by the beneficial effect of this substance on MAPK, NF-κB and PI3K but also by its influence on the monoaminergic system. The use of a 5HT7 antagonist abolished the effect caused by fisetin, and the administration of a serotonin precursor enhanced it [98]. Moreover, fisetin reduced the depressive and anxiety symptoms that co-occurred with neuropathic pain [98]. It was also shown that the blockade of spinal GABAA receptors by bicuculline completely counteracted fisetin analgesia, so these receptors are probably an indirect target for this substance [99]. There are also data from studies conducted in a model of diabetic neuropathy in rats showing that the oral administration of fisetin alleviated hypersensitivity, which is associated with reduced levels of NF-κB and is probably a consequence of pronociceptive IL-6 and TNFα [100]. Moreover, it has also been determined that fisetin positively modulates the activity of microglia; in vitro studies have shown that this substance inhibits the expression of iNOS, and, as a consequence, the production of NO and IL-1β; the regulatory molecular mechanism of fisetin-induced HO-1 expression operates through the PI3K/AKT and p38 signaling pathways [94]. The suggested factors important in nociception affected by fisetin are presented in brief in Scheme 4. Importantly, the latest data suggest that fisetin may have a positive effect on other states that may result in neuropathic pain development; for example, an in vitro study showed that this substance acts as a growth inhibitor of human oral squamous cell carcinoma [230]. Additionally, there are also reports which indicate that fisetin exerts antihyperlipidemic [231] and antidiabetic [232] properties. Understanding the molecular mechanisms of fisetin is essential to develop new, safe, and effective strategies for the treatment of neuropathic pain. The improvement in the effectiveness of opioid treatment after combined administration with fisetin, demonstrated in experimental studies, opens new possibilities for combined therapy. 

## 11. Conclusions

The research on the selected multitarget substances discussed above indicates that both MAPKs and factors such as NRF2, NF-κB, PI3K and MMP9 may become important targets for future neuropathic pain therapies. This notion is supported by pharmacological studies conducted with animal neuropathic pain models using selective and nonselective pharmacological tools. Further research is clearly needed, as the modulation of intracellular pathways underlying many pathophysiological processes may also improve the effectiveness of analgesics that are already in clinical use. In our opinion, neuropathic pain therapy should be based on nonselective drugs with a broad spectrum of action, which, by partially inhibiting selected intracellular pathways strongly activated in neuropathy, will be able to at least partially restore homeostasis. In our opinion, such valuable substances include minocycline, astaxanthin, fisetin, and peimine, which were considered to be effective in pain relief in behavioral studies; their beneficial properties result from their wide spectrum of action, among others, on various intracellular pathways. Importantly, these substances exert suitable analgesic properties in neuropathy models of various etiologies and are also relatively safe for patients because some are already used in the clinic, not to relieve pain, but, for example, in the case of minocycline. in the treatment of bacterial infections, acne, or rheumatoid arthritis. Formulations containing fisetin or astaxanthin are also broadly available to buy as dietary supplements with antioxidant properties. It is worth emphasizing that the results of behavioral tests also suggest their usefulness in therapy combined with opioid drugs, the effectiveness of which decreases under neuropathic pain. We believe that future research on these compounds should focus on a detailed understanding of the mechanisms of action of these substances, particularly in the context of gender differences in different phases of neuropathic pain. Moreover, these studies, conducted on various neuropathy models, should also take into account the ability of these substances to alleviate neuropathic pain comorbidities (e.g., metabolic, autoimmune, viral or cancer diseases). Overall, a detailed understanding of the pharmacokinetic profile and the impact of these compounds on pathophysiological processes may contribute to the development of precise and effective treatments for neuropathic pain, taking into account the individual condition of patients.

## Figures and Tables

**Table 1:** Summary of suggested targets of minocycline, astaxanthin, fisetin, and peimine and their influence on important factors in nociception and on the effectiveness of opioid treatment in various animal models of neuropathic pain.

NEUROPATHIC PAIN				
SUBSTANCE	SUGGESTED DIRECT TARGETS	NEUROPATHIC PAIN ANIMAL MODELS	INFLUENCE ON IMPORTANT FACTORS IN NOCICEPTION	EFFECT ON OPIOID TREATMENT EFFECTIVENESS
**Minocycline**	**p38** [42,70,71] **ERK** [72] **MMP9** [73]	**CCI** (mouse) [74,75] **CCI (rats)** [76,77] **SCI (rats)** [78] **STZ (rats)** [79] **SNL (rats)** [70] **STZ (mouse)** [80]	**iNOS ↓** [81] **IL-1β** [82] **IL-6 ↓** [26] **IL-18 ↓** [26] **MMP9 ↓** [26] **MMP2 ↓** [26] **XCL1 ↓** [83] **pERK ↓** [72,84] **PI3K ↓** [84]	**morphine ↑** [26,74]
**Astaxanthin**	**p38** [85] **ERK** [85] **NF-κB** [86] **NR2B** [87] **ROS** [88]	**SNL (mouse)** [85] **CCI (rat)** [89] **SCI (rat)** [87] **CCI (mouse)** [90]	**IL-1β ↓** [85] **IL-6 ↓** [85] **IL-4 ↑** [85] **IL-10 ↑** [85] **pERK ↓** [85] **pp38 ↓** [85] **NF-κB ↓** [85]	**morphine ↑** [90] **buprenorphine ↑** [90] **oxycodone ↑** [90]
**Peimine**	**p38** [91] **ERK** [92] **JNK** [92] **NF-κB** [92] **Kv1.3** [93] **Nav1.7** [93]	**CCI (mouse)** [90]	__________	**morphine ↑** [90]
**Fisetin**	**p38** [94] **ERK** [95] **Pi3K** [96] **NF-κB** [97] **5HT7** [98] **GABA** [99]	**CCI (mouse)** [98] **CCI (mouse)** [90] **STZ (mouse)** [99] **STZ (rat)** [100]	**IL-6 ↓** [100] **TNFα ↓** [100] **NF-κB↓** [100] **NRF2 ↑** [100]	**morphine ↑** [90] **oxycodone ↑** [90]

**Abbreviations:** spinal nerve ligation model (SNL); chronic constriction injury of the sciatic nerve model (CCI); spinal cord injury model (SCI), streptozotocin-induced diabetic model (STZ), interleukin (IL); inducible nitric oxide synthase (iNOS); chemokine-C-motif ligand 1 (XCL1); tumor necrosis factor alpha (TNFα); reactive oxygen species (ROS); complement component 1q (C1q); microglia/macrophage markers (Iba, CD11b/c), astroglia marker (GFAP); matrix metalloproteinases (MMP); p38 Mitogen-Activated Protein Kinase (p38MAPK); Extracellular signal-regulated kinases (ERK); phosphoinositide 3-kinase (PI3K); Nuclear factor kappa B (NF-κB); glutamate N-methyl-D-aspartate receptor subunit NR2B (NR2B); 5-hydroxytryptamine 7 receptor (5-HT7); Gamma-Aminobutyric Acid receptor (GABA); Nuclear factor erythroid 2-related Factor 2 (NRF2); ↓decrease; ↑ increase.

## Data Availability

Data sharing is not applicable.
